# Supplementary material for: Functional partitioning of lipoic acid decouples cellular abundance from mitochondrial utilization
Source: J Lipid Res. 2026 Aug 20;67(9):101126. doi: 10.1016/j.jlr.2026.101126 (PMC13602143; doi:10.1016/j.jlr.2026.101126)

Supplemental Figure 1

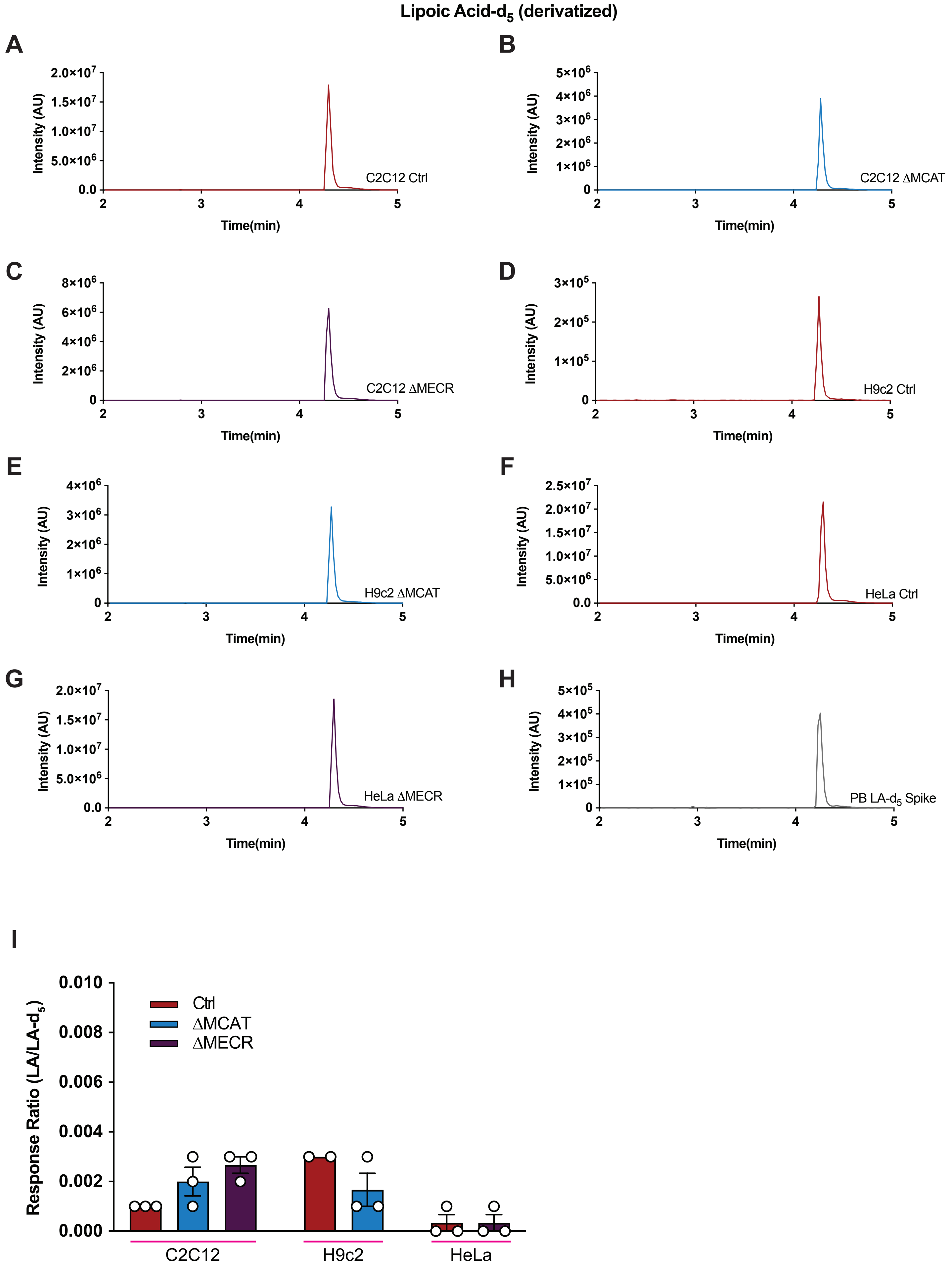

Supplement: Supplementary file 1 [file mmc1.pdf]
